# Control of mRNA fate by its encoded nascent polypeptide

**DOI:** 10.1016/j.molcel.2023.07.014

**Published:** 2023-08-17

**Authors:** Markus Höpfler, Ramanujan S. Hegde

**Affiliations:** 1 MRC Laboratory of Molecular Biology, Cambridge, UK

**Keywords:** protein biogenesis, mRNA decay, nascent chain, mRNA localization, translational regulation, ribosome

## Abstract

Cells tightly regulate mRNA processing, localization, and stability to ensure accurate gene expression in diverse cellular states and conditions. Most of these regulatory steps have traditionally been thought to occur before translation by the action of RNA-binding proteins. Several recent discoveries highlight multiple co-translational mechanisms that modulate mRNA translation, localization, processing, and stability. These mechanisms operate by recognition of the nascent protein, which is necessarily coupled to its encoding mRNA during translation. Hence, the distinctive sequence or structure of a particular nascent chain can recruit recognition factors with privileged access to the corresponding mRNA in an otherwise crowded cellular environment. Here, we draw on both well-established and recent examples to provide a conceptual framework for how cells exploit nascent protein recognition to direct mRNA fate. These mechanisms allow cells to dynamically and specifically regulate their transcriptomes in response to changes in cellular states to maintain protein homeostasis.

## Teaser

Accurate gene expression depends critically on mRNA processing, localization, stability, and translation. Höpfler and Hegde review the myriad ways that a nascent protein can co-translationally regulate the usage and fate of its encoding mRNA. These dynamic feedback mechanisms control many aspects of cellular physiology and can go awry in disease.

## Introduction

The features of a messenger RNA (mRNA) molecule ultimately dictate the quality and quantity of proteins translated from it. In eukaryotic cells, mRNA is transcribed from DNA in the nucleus and undergoes a series of essential processing steps prior to its export to the cytoplasm. The major processing reactions include 5′ capping, 3′ polyadenylation, various internal base modifications, and splicing. Transcription, processing, and nuclear export are all highly regulated to determine the amount and sequence of each mRNA available to the cytoplasmic translation machinery.^1^^,^^2^^,^^3^^,^^4^

In the cytoplasm, multiple layers of post-transcriptional regulation ensure that mRNAs are translated at the right time, in the right place, and at the appropriate rate to produce the desired amount of protein. The best-studied regulatory motifs are *cis*-acting signals in the mRNA. Such signals can be recognized by sequence- or structure-specific RNA-binding proteins or complementary RNA molecules, such as micro-RNAs.^5^^,^^6^ These recognition events are then coupled to factors that can control mRNA translation, stability, modifications, or localization. Each of these mechanisms is widely used for both general and highly targeted regulation.

**Figure 1:**
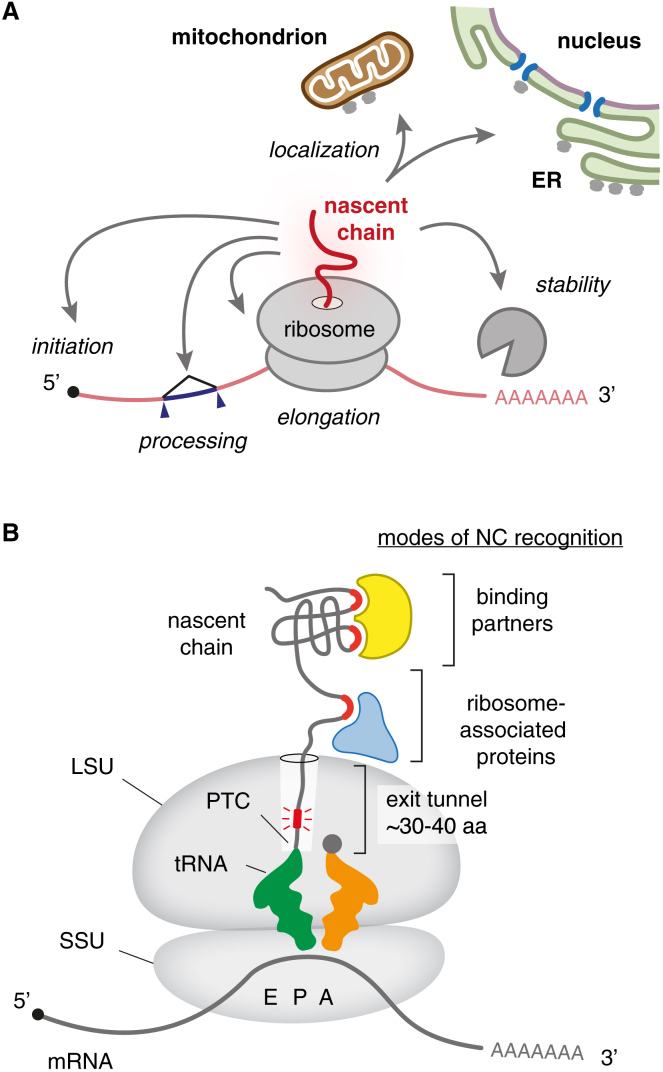
The nascent protein chain regulates mRNA fate (A) The nascent protein chain emerging from a ribosome is physically coupled to its encoding mRNA during translation. This coupling allows the nascent chain to regulate several aspects of the fate and usage of its mRNA: translation initiation, elongation, mRNA processing, stability, and localization. (B) Specificity during nascent chain-mediated regulation of its mRNA requires recognition of specific sequence motifs (red) by one of two modes. The first mode of recognition is mediated by the ribosome and occurs inside its exit tunnel, which houses ∼30–40 amino acids (aa) of the nascent chain. The second mode of recognition is mediated by *trans*-acting factors that include ribosome-associated proteins (blue) and nascent chain binding partners (yellow). These recognition events trigger a series of downstream effects that influence mRNA fate, often working through additional effectors (not shown). Black dot marks the 5′ mRNA cap. Ribosomal tRNA binding sites are marked: A, amino-acyl site (orange tRNA); P, peptidyl site (green tRNA); E, exit site (tRNA not shown). LSU/SSU, large/small ribosomal subunit; PTC, peptidyl-transferase center.

A less-appreciated mechanism of mRNA regulation occurs co-translationally. In these cases, the sequence or structure of the nascent protein chain (hereafter “nascent chain,” or NC) serves as the specificity element for recognition. As with mRNA *cis*-regulatory motifs, the NC makes interactions with *trans*-acting factors (or the ribosome itself) to ultimately impact mRNA translation, localization, processing, and stability—collectively termed “mRNA fate” (Figure 1A). A classic example is the localization of secretory protein mRNAs to the surface of the endoplasmic reticulum (ER) via co-translational recognition of N-terminal signal sequences.^7^

Here, we review established and newly emerging mechanisms of NC-triggered regulation of mRNA fate. We argue that NC recognition is an important complement to *cis*-acting mRNA sequence motifs, which together regulate mRNA fate, and ultimately protein expression, to control numerous aspects of protein homeostasis. Although the breadth and diversity of NC-directed mRNA regulation are not known, the increasingly large number of ribosome- and NC-binding factors hints at a rich and widespread mode of regulation.^8^

## The NC emerges into a crowded environment

A protein’s folding and maturation begin during its synthesis at the ribosome^9^^,^^10^ (Figure 1B). These maturation events involve intramolecular and intermolecular interactions of the NC. New amino acids are added at the peptidyl-transferase center (PTC) in the large ribosomal subunit (LSU). From here, the NC passes through a long ∼100 Å exit tunnel in the large subunit. The first interactions made by the NC are with the walls of this tunnel, comprising mostly the ribosomal RNA (rRNA) backbone and portions of a few ribosomal proteins.^9^^,^^10^^,^^11^

As the NC approaches the mouth of the exit tunnel, it begins making interactions with non-ribosomal proteins, some of which can even probe part of the tunnel.^12^ A large number of proteins can access the NC once it begins emerging into the cytosol. These include chaperones,^13^ modification enzymes,^14^ targeting factors,^15^ processing factors such as proteases,^16^ co-factors,^17^ interaction partners,^18^^,^^19^ and even other NCs.^20^ Many of these factors can bind directly to the ribosome near the tunnel exit, whereas others bind only via the NC^21^ (Figure 1B). All of these interactions participate in the protein’s eventual maturation to a functional product. Each interaction is therefore an indirect indicator of where along the maturation pathway a protein is; conversely, failure of one or another interaction, or an inappropriate interaction, can be an indicator of aberrant biogenesis.

Through these interactions, the cell can monitor the biogenesis status of a protein during its translation. Given the central role of protein homeostasis to numerous aspects of cell fitness,^22^^,^^23^ several feedback mechanisms have evolved to convey a protein’s biogenesis status to the encoding mRNA. By modulating that mRNA’s fate, the cell is able to control the location, rate, and duration of protein production in response to a change in that protein’s biogenesis. Feedback mechanisms linking the NC to its mRNA are widespread across biology, from relatively simple prokaryotes to multicellular organisms. All of these mechanisms occur co-translationally because this is the one period in every protein’s biogenesis when the protein and mRNA are physically linked, thereby facilitating effects in *cis*. The diversity of NC interactions provides numerous potential opportunities for this type of regulation, only a subset of which have probably been uncovered.

## Two modes of NC feedback on its mRNA

The mouth of the exit tunnel where the NC emerges into the cytosol is very far from the entrance and exit of the mRNA channel that runs through the small ribosomal subunit (SSU). For this reason, the NC typically does not directly contact its encoding mRNA. Instead, NC-mediated effects on the fate of its encoding mRNA operate via intermediaries—either the ribosome or *trans*-acting factors that bind to the NC, mRNA, or ribosome. Thus, NC-mediated effects involve an NC recognition event and downstream effector(s) that influence mRNA fate.

There are two potential mechanisms by which the NC can impact mRNA fate. They are not mutually exclusive and can sometimes operate in combination. This section introduces the two mechanisms in general terms, with the remainder of the review delving into the many variations of each mechanism by drawing on specific examples.

The first mechanism involves NC interactions with the ribosome to impact translation elongation or termination. The NC-ribosome interaction occurs inside the tunnel, typically close to the PTC where an incoming aminoacyl-tRNA adds the next amino acid to the growing polypeptide. NC interactions with the ribosome tunnel can propagate conformational changes to the peptidyl-tRNA or PTC to impair peptidyl transfer (or the analogous termination reaction). This not only impacts mRNA translation efficiency but can have further consequences for mRNA stability, processing, or localization as described later.

The second mechanism involves recognition of the NC by *trans*-acting factors that directly or indirectly influence mRNA fate. Recognition can be exquisitely specific to a particular amino acid sequence or structure or can involve more widely occurring elements such as signal sequences. The *trans*-acting factor, typically in combination with additional factors, can influence various aspects of mRNA fate. As noted above, the two mechanisms are not mutually exclusive and can sometimes act in concert. For example, stalling of translation elongation via NC-ribosome interactions (i.e., mechanism 1) can be relieved by a *trans*-acting factor that engages the NC (i.e., mechanism 2), thereby allowing regulatory control of stalling and its downstream consequences.

## NC-ribosome interactions stall translation

**Figure 2:**
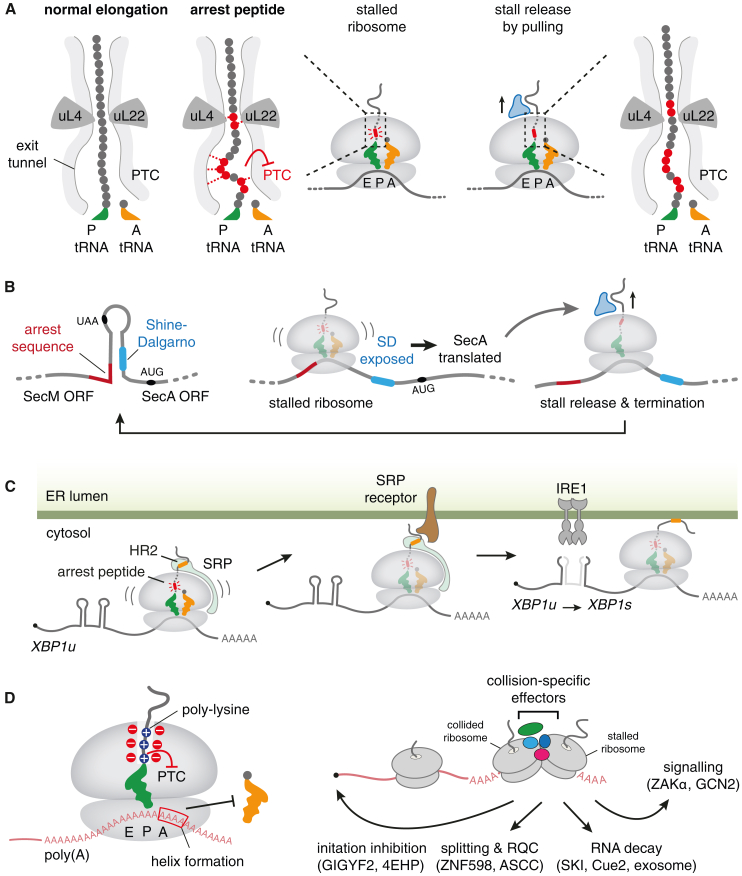
Regulation of mRNA fate by nascent chain stalling (A) During normal translation elongation, individual amino acids in the nascent chain (gray dots) do not make stable contacts with the ribosome exit tunnel (left diagram). By contrast, a subset of its amino acids in arrest peptides form interactions with the ribosome exit tunnel (red dots with dashed lines in the middle diagrams). These interactions typically form in the region of the exit tunnel between the PTC and a constriction site formed by uL22 and uL4. The interactions can distort the peptidyl-transferase center (PTC) or induce nascent chain secondary structure to alter the geometry of the peptidyl-tRNA. The consequence is an impairment of peptidyl transfer from the P-site tRNA (green) to the incoming aminoacyl-tRNA (orange). Arrest peptides can be released by factors that impart a pulling force, thereby breaking ribosome-nascent chain interactions (right diagram). (B) Example of how an arrest peptide can regulate translation from its encoding mRNA. The SecA ORF downstream of the SecM ORF is normally prevented from being translated because its Shine-Dalgarno (SD) sequence is part of a stem-loop. When SecM is translated and stalls at an arrest peptide, the ribosome disrupts this stem-loop, exposing the downstream SD to allow SecA translation. After sufficient SecA (blue) is produced, it can engage the SecM nascent chain and mediate its targeting to the periplasmic membrane (not shown), where a pulling force during SecM translocation into the periplasm relieves the stall. Ribosomes that do not stall on SecM elongate further and terminate, which allows re-folding of the stem-loop structure to attenuate SecA translation. (C) Ribosome stalling induced by the nascent chain can impact mRNA localization and processing. Left: translation slow-down by an arrest peptide in unspliced *XBP1* mRNA (termed *XBP1u*) facilitates recognition of a weak signal sequence (hydrophobic region 2 [HR2]) by the signal recognition particle (SRP). Center: this leads to targeting of the ribosome-nascent-chain complex to the endoplasmic reticulum (ER) membrane via the SRP receptor. Right: targeting of *XBP1u* mRNA to the ER surface improves access to IRE1, which mediates splicing of a short intron from *XBP1u* to produce the spliced isoform (*XBP1s*) during the UPR. *XBP1s* encodes for an active transcription factor that controls downstream effector genes. (D) Ribosome stalling on the poly(A) tail of non-stop mRNAs triggers a multi-faceted response. The mechanism of stalling (left) involves a combination of slowed elongation due to interactions of the poly-lysine peptide (encoded by AAA codons) with the negatively charged tunnel surface and unfavorable helical formation of the poly(A) sequence in the A-site that impairs decoding. Stalling of a ribosome on poly(A) leads to collision with a trailing ribosome (right). The compound surface of the collided disome serves as a platform to recruit collision-specific effectors that mediate a range of outcomes as indicated by the arrows: (1) cap-dependent translation initiation inhibition by GIGYF2 and 4EHP; (2) collision dissociation and ribosome-associated quality control (RQC) by ZNF598, ASCC, and other factors; (3) mRNA decay by the SKI complex, Cue2, and the exosome; and (4) stress signaling via the kinases ZAKα and GCN2.

Even before leaving the ribosomal exit tunnel and interacting with non-ribosomal proteins, nascent peptide sequences can have a profound impact on the fate of the encoding mRNA. The effects are typically mediated by interactions of the NC with the ribosome exit tunnel, causing translation to stall (Figure 2A). These elongation-stalling elements are termed arrest peptides, although in many cases, the effect is a kinetic slowdown and not a permanent arrest.^11^^,^^24^ Arrest peptides can be sequence-specific elements, typically around 10–15 amino acids, or short stretches of polypeptide that possess a general biophysical property, such as hydrophobicity or positive charge.

Numerous structural studies of ribosomes stalled by sequence-specific or non-specific arrest peptides show that the ribosomal exit tunnel can constrain the NC to particular conformations or even induce NC secondary structure formation.^25^^,^^26^^,^^27^^,^^28^^,^^29^ These interactions typically occur within the proximal segment of the tunnel prior to a constriction site between ribosomal proteins uL4 and uL22 (Figure 2A). NC-ribosome interactions can impair translation elongation (or termination) by either distorting the PTC or altering the geometry of the peptidyl-tRNA bond. In both cases, the incoming aminoacyl-tRNA (or release factor in the case of termination) is disfavored from reacting with the ester bond of the peptidyl-tRNA, thereby stalling translation.

The numerous specific ways arrest peptides distort the PTC, or peptidyl-tRNA are reviewed elsewhere.^11^^,^^24^ For the purposes of this review, the salient point is solely that NC-ribosome interactions impede the chemistry of elongation or termination at the PTC. In the following examples, we focus on how cells have evolved to exploit arrest peptides to modulate expression of co-linear genes, control translation rates of the encoding mRNA, localize the encoding mRNA, regulate mRNA processing, or trigger mRNA decay.

## Regulation of gene expression by arrest peptides

Arrest peptides were first characterized in prokaryotes, where their analyses revealed several general principles of NC-mediated gene regulation.^11^^,^^24^ The major concept that has emerged from prokaryotic systems is that translation arrest is a mechanism to regulate production of one or more proteins from that operon under a subset of physiologic conditions. This regulation is accomplished by the arrested ribosome, which occludes a ∼25–30 nucleotide stretch of mRNA, locally influencing mRNA structure and accessibility.^30^^,^^31^ Translation initiation or mRNA processing reactions in the region of the stalled ribosome are altered, thereby influencing gene expression.

In one of the first and best understood examples, a 17 amino acid arrest peptide in bacterial SecM (for “secretion monitor”) triggers ribosome stalling near the end of its open reading frame (ORF).^32^ A ribosome stalled at this site induces a local change in mRNA secondary structure such that an immediately downstream Shine-Dalgarno (SD) translation start site for the next ORF is available for initiation (Figure 2B).^33^ This downstream ORF encodes for SecA, an ATPase involved in protein secretion.^34^ Once the cell has produced sufficient SecA to accommodate its secretion needs, it is available to engage the SecM nascent protein and target it to the plasma membrane, where a pulling force induced by SecM translocation relieves the stall.^35^ When the stall is relieved, the ribosome proceeds to the stop codon, terminates, and the mRNA secondary structure refolds to occlude the SD sequence of the downstream SecA, thereby attenuating its translation.^32^ Thus, in this autoregulatory system the SecM NC directly regulates translation initiation from its encoding mRNA to control SecA levels, which, in turn, modulates SecM translation elongation.

A second illustrative prokaryotic example is TnaC.^36^^,^^37^^,^^38^ Here, the arrest peptide is located at the C terminus of a 24-amino-acid leader peptide and stalls translation at the stop codon by inhibiting termination. Importantly, this arrest peptide only engages the ribosome tunnel in a ternary complex with free L-tryptophan, highlighting the principle of metabolite-induced arrest.^28^^,^^29^^,^^36^ The arrested ribosome, which trails RNA polymerase during co-transcriptional translation in bacteria, covers a segment of nascent mRNA that would otherwise form a hairpin structure.^38^ This hairpin normally triggers transcriptional termination of the adjacent RNA polymerase; therefore, a stalled ribosome at this site allows transcription to continue. This generates a longer mRNA that now contains two downstream ORFs coding for TnaA and TnaB, proteins involved in tryptophan metabolism. When tryptophan levels decline through the action of these proteins, arrest no longer occurs, resulting in transcriptional termination upstream of the *tnaA* and *tnaB* genes. Thus, the NC influences the processing of its encoding mRNA, thereby determining what is encoded by the mRNA.

These examples, among many similar ones in prokaryotes,^11^^,^^24^ illustrate two key general principles of arrest peptides. First, arrest can be used to regulate the identity and usage of the encoding mRNA. Although both of these examples rely on prokaryote-specific biology (operons and co-transcriptional translation), the concept of an arrested ribosome serving as a platform for initiating (or inhibiting) a downstream reaction has emerged as a general mechanism in eukaryotes as elaborated below. Second, arrest peptides need not constitutively arrest translation; instead, their action is context-dependent and can be modulated by *trans*-acting proteins or small molecules.

Despite the absence of operons in eukaryotes, analogous types of arrest-mediated gene regulation have been described in two contexts. First, many viruses that infect eukaryotic cells encode multiple proteins from one mRNA. Translational arrest at the boundaries between viral proteins can provide a means of regulating the downstream translation products. Indeed, one of the few characterized eukaryotic arrest peptides comes from an upstream ORF (uORF) in human cytomegalovirus where stalling at the stop codon of this uORF precludes expression of the downstream glycoprotein.^39^^,^^40^ The second example, from the yeast *Neurospora crassa*, similarly involves stalling at a short uORF that encodes a 24-amino-acid protein termed the arginine attenuator peptide (AAP). Stalling by AAP, which is dependent on free L-arginine (analogous to the bacterial TnaC system), inhibits translation of the primary ORF encoding a key subunit of an arginine metabolism enzyme.^41^^,^^42^ It is noteworthy that the major ORF in almost half of all mammalian mRNAs is preceded by one or more uORFs that can potentially modulate expression of the main ORF.^43^^,^^44^ Some of these cases might involve NC-mediated arrest in a context-dependent manner as exemplified by AAP.

## Arrest peptides facilitate mRNA localization

One consequence of translation arrest is that the NC spends a longer time at a specific length. The binding of *trans*-acting factors that engage the NC at that length is therefore favored. Hence, an arrest peptide acting inside the ribosome tunnel can influence which factors are recruited to the NC outside the ribosome. The recruited factor is then in a position to regulate the fate of the ribosome-associated mRNA. This principle of arrest-mediated factor recruitment is used during translation of mammalian *XBP1* mRNA (Figure 2C), an important mediator of the mammalian unfolded protein response (UPR).

The UPR is a set of signaling pathways that communicate protein folding stress within the ER to the transcription and translation machinery.^45^^,^^46^ One pathway is initiated by IRE1, a transmembrane protein that is activated by protein misfolding stress in the ER and signals gene expression changes in the nucleus. In mammalian cells, activated IRE1 mediates endonucleolytic cleavage of a 26-nucleotide intron from *XBP1* mRNA.^47^^,^^48^ Joining the ends of cleaved *XBP1* mRNA by a ligase^49^ produces a processed version of *XBP1* mRNA that now codes for a transcription factor that activates stress-response genes.

Splicing of *XBP1* mRNA is influenced by two distinct features of the encoded NC: a hydrophobic region (HR) (termed hydrophobic region 2 [HR2]) and a downstream arrest peptide^50^^,^^51^ (Figure 2C). HR2 engages the signal recognition particle (SRP), a targeting factor for delivering ribosome-NC-mRNA complexes to the ER.^52^^,^^53^ However, this process is inefficient for XBP1 because the moderate hydrophobicity of HR2 makes it a suboptimal SRP substrate. Recognition by SRP is improved by the downstream arrest peptide, which stalls the ribosome just after HR2 emerges from the ribosomal exit tunnel.^50^^,^^51^ The combined action of HR2 and the arrest peptide facilitates SRP-mediated targeting of the ribosome-NC-mRNA complex to the ER, thereby localizing the mRNA. Localization of *XBP1* mRNA improves its access to and cleavage by IRE1, enhancing the efficiency of UPR signaling. *XBP1* therefore provides a paradigm for how an arrest peptide facilitates localization and processing of its encoding mRNA.

## Ribosome stalling, mRNA decay, and quality control

In addition to sequence-specific arrest peptides, other properties of the NC can impact translation from within the ribosome exit tunnel. The best characterized of these is the interaction of a poly-basic segment of the NC with the negatively charged surface of the ribosomal exit tunnel. Although such segments have long been known to slow translation,^54^^,^^55^ the physiologic consequences for such stalling have only become clear in recent years. The best understood function of translation stalling by poly-basic sequences in eukaryotes is for the detection of aberrant mRNAs that are inappropriately polyadenylated in the coding region at a near-cognate polyadenylation signal. This can result in a truncated mRNA without an in-frame stop codon (often termed a non-stop mRNA). Mis-polyadenylation is thought to be a common mRNA processing error due to the frequency of near-cognate polyadenylation signals.^56^^,^^57^

Elimination of non-stop mRNAs is translation dependent.^56^ Translation of the poly(A) sequence produces poly-lysine, which slows translation due to NC-tunnel interactions that impede peptidyl-transfer^58^^,^^59^ (Figure 2D). When translation slows, the poly(A) mRNA in the A site of the ribosome has time to adopt a helical configuration that is incompatible with decoding, effectively stalling translation.^58^^,^^59^ The next ribosome of the polysome eventually collides with the stalled ribosome. Collided ribosomes have a distinctive structure and can be recognized by several factors that trigger different downstream consequences, including translation inhibition, mRNA decay of the associated mRNA, and initiation of stress signaling pathways (Figure 2D; for recent reviews, see Yip and Shao,^60^ D’Orazio and Green,^61^ Kim and Zaher,^62^ and Filbeck et al.^63^).

The inhibition of mRNA translation initiation in eukaryotes is mediated by the GIGYF2-4EHP complex.^64^^,^^65^^,^^66^ Stable binding of the GIGYF2-4EHP complex to collided ribosomes relies to a large extent on EDF1, a collision-specific binding protein.^64^^,^^65^ Once recruited, 4EHP (also called EIF4E2) can bind to the 5′ cap on the mRNA, thereby preventing the initiation factor eIF4E from binding to the cap.^64^^,^^65^ By preventing initiation of new ribosomes, the GIGYF2-4EHP complex would provide time for the collision(s) to be resolved without generating even longer ribosome queues.

The E3 ubiquitin ligase ZNF598 (Hel2 in yeast) is another eukaryotic collision sensor that is recruited along with the GIGYF2-4EHP complex. If the collision persists, ZNF598 eventually monoubiquitinates ribosomal protein eS10 (or uS10 in yeast).^67^^,^^68^ This marks the stalled ribosome-mRNA complexes for multiple downstream reactions, the order and coordination of which are not well understood. These reactions include ribosome splitting by the ASCC helicase complex,^69^^,^^70^ endonucleolytic cleavage of the mRNA by Cue2/NONU-1,^71^^,^^72^ and 3′ to 5′ exonuclease degradation of the mRNA by the SKI helicase and associated cytoplasmic RNA exosome.^73^ Thus, a poly-lysine NC segment participates in translation inhibition and can eventually lead to degradation of specifically its encoding non-stop mRNA.

It is currently thought that the events downstream of ribosome collisions are independent of the mechanism that triggered the stall. Hence, stalls on mRNAs with UV damage or extensive secondary structure similarly lead to ribosome collisions and its downstream consequences.^74^^,^^75^^,^^76^ The implication of this is that physiologic stalls mediated by strong arrest peptides could also modulate translation initiation rates or mRNA stability. Because such stalls can be modulated by *trans*-acting factors or even small molecules as exemplified above, there may exist undiscovered examples of regulated mRNA decay triggered by stalls induced by the encoded NC.

Consistent with this idea, poly-proline tracts and certain other motifs that have the potential to stall translation are normally resolved by eIF5A in eukaryotes, which improves peptide-bond formation.^77^^,^^78^^,^^79^^,^^80^^,^^81^ Hence, poly-proline peptides normally do not destabilize their associated mRNAs.^82^ However, if certain stress or physiologic conditions were to inactivate eIF5A, a subset of mRNAs could be preferentially destabilized or translationally inhibited in an NC-mediated manner. Similarly, a recent study in human cells showed that specific motifs containing di-peptide repeats with a positively charged and a bulky residue or peptides with high β strand propensity can slow ribosomes *in vitro* and destabilize the encoding transcripts in cells.^82^ A range of distinct factors appear to be involved in the decay of stall-inducing mRNAs,^83^^,^^84^^,^^85^ providing ample opportunity for substrate-specific regulation of mRNA stability.

## How frequent is NC-triggered arrest?

Transcriptome-wide analyses in different eukaryotic systems have been performed to identify sites of ribosome collisions, mRNA mis-adenylation, and increased ribosome occupancy.^31^^,^^57^^,^^86^^,^^87^^,^^88^^,^^89^ These studies do not directly monitor the frequency of NC-mediated arrest because many sites of stalling might be due to mRNA features such as codon usage or secondary structure. Furthermore, not all arrest events would lead to collisions, and some arrest events might only be observed during particular physiologic states. Nonetheless, two provisional conclusions can be drawn.

First, the frequency of stalling by mRNA sequence-independent NC elements might be high. One source of such stalls might be mis-adenylation at near-cognate polyadenylation sites, an error that is thought to be pervasive.^56^^,^^57^^,^^87^ Hence, poly(A)-triggered stalling is likely to be prevalent, perhaps explaining why loss of factors involved in its resolution leads to protein misfolding stress in yeast cells^90^^,^^91^^,^^92^ and embryonic lethality in mice.^93^^,^^94^ A second potential source of stalling might be a hydrophobic segment, such as a transmembrane domain, located inside the ribosome. This has been shown for one case^95^ and implicated in others in both yeast and mammalian systems.^86^^,^^96^ Such stalls might normally be very subtle if they are rapidly relieved when protein biogenesis proceeds normally, but they could become prevalent under conditions of failed biogenesis, such as protein misfolding or stress.^96^ Consistent with this idea, conditions of limited chaperone availability, such as acute protein misfolding stress, lead to widespread stalling of NCs at a point where they are just emerging into the cytosol in mammalian cells.^97^^,^^98^

Second, it is very difficult to identify sequence-specific arrest peptides from the types of analysis performed so far. The reasons for this are 2-fold. First, arrest peptides cannot be predicted based on primary sequence using the known examples, each of which differs substantially from one another. Second, even if the arrest is strong, it might be quickly relieved under most experimental conditions. These challenges might explain why only a few sequence-specific arrest peptides are well established. An important challenge is to find systematic ways of reliably identifying arrest peptides, the characterization of which might lead to the discovery of new modes of regulation.

## Arrest peptides in research and therapeutics

The ability to modulate arrest in *trans* not only provides a regulatory mechanism for the cell but has also been exploited as a research tool. For example, the ability of the SecM arrest peptide to be relieved by a pulling force has been used to generate reporters that detect co-translational forces on an NC.^99^^,^^100^ These reporters have revealed that forces are experienced by the NC when it folds at the mouth of the ribosome exit tunnel^101^ or when it partitions into a membrane.^99^ Such forces are transient and weak, making them otherwise very challenging to detect. Because arrest peptides from different proteins (or mutant variants) are relieved by different amounts of force,^27^^,^^102^ they might have relatively broad versatility in detecting co-translational processes occurring at or near the ribosome surface.

Arrest peptides can be potentially harnessed for therapeutic purposes if stalling by specific NCs can be induced by a small molecule. This is analogous to how tryptophan is an essential co-factor in stalling of the TnaC arrest peptide.^36^ A proof of concept for this idea has been demonstrated for translation inhibition of mammalian PCSK9, a therapeutic target that regulates plasma levels of low-density lipoprotein.^103^^,^^104^ A small molecule has been shown to bind co-translationally to a specific sequence in the PCSK9 NC only when it resides at a particular part of the ribosome exit tunnel. The ternary complex of the ribosome exit tunnel, small molecule, and PCSK9 stabilizes an NC conformation that impedes peptidyl transfer, ultimately leading to lower PCSK9 protein production. Similarly, it was shown that the compound can selectively inhibit translation termination when the stop codon is preceded by certain amino acid combinations.^104^^,^^105^ Thus, modulating translation elongation or termination of specific NCs represents a potentially productive avenue for drug discovery.

## NC recognition at the ribosome exit tunnel

As the NC emerges into the cytosol, it can be recognized by factors that range widely in their specificity. Some factors, such as methionine aminopeptidase or the NC-associated complex (NAC), are so general that they sample nearly all NCs.^14^^,^^21^ Other factors, such as SRP, are specific for certain classes of substrates such as secretory and membrane proteins.^106^^,^^107^ And some factors are exquisitely specific for only one or a handful of substrates.^108^ Collectively, NC-binding factors have numerous roles in protein biogenesis ranging from modifications, folding, localization, proteolytic processing, and others.

As described in the following sections, a subset of NC-binding proteins regulate either the localization or stability of the associated mRNA. These effects on mRNA fate rely critically on co-translational recognition at the ribosome, which is the only time when the NC and its encoding mRNA are physically linked. Selective co-translational recognition relies on two distinctive features of nascency. First, the polypeptide is not yet folded into its final structure, exposing surfaces that are buried in the mature protein. Second, the translating ribosome provides a platform to allow binding factors to recognize a composite surface formed by the ribosome and NC. Thus, coincidence detection is a common mechanism for achieving selectivity of NC recognition at the ribosome.

## mRNA localization to intracellular membranes

**Figure 3:**
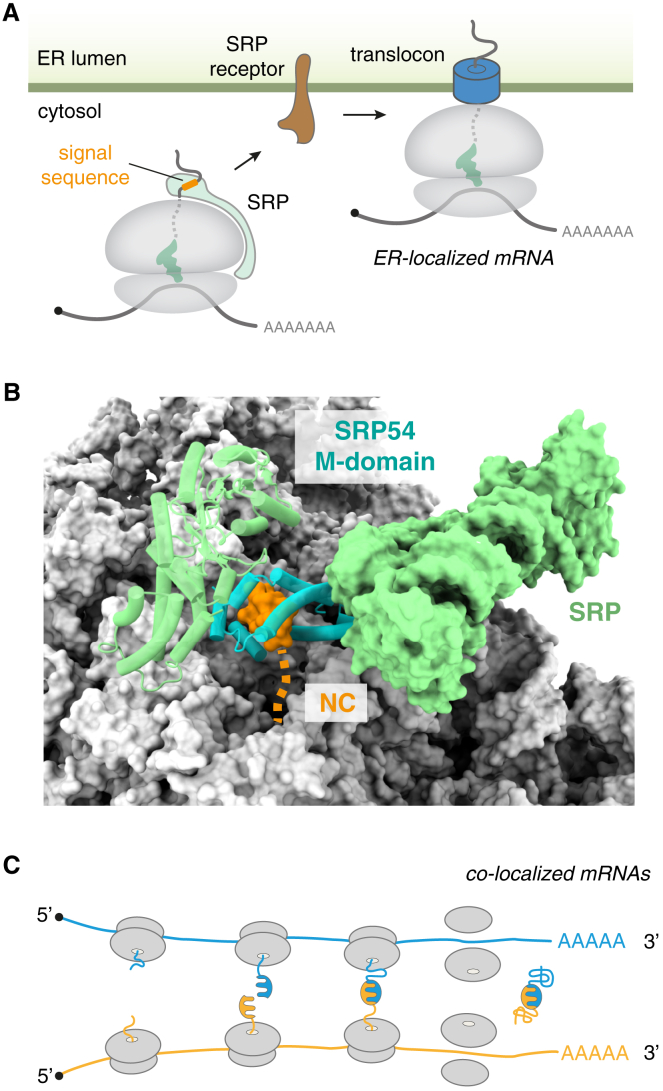
Nascent chain-mediated mRNA localization (A) When a hydrophobic signal sequence (or transmembrane domain) is translated, the signal recognition particle (SRP) is recruited to the ribosome. This leads to targeting of the ribosome-nascent chain complex with the associated mRNA to the ER (or plasma membrane in prokaryotes) via an interaction between SRP and the SRP receptor. The ribosome docks on a translocon centered around Sec61 (or SecY in prokaryotes), thereby localizing the mRNA. (B) The SRP54 M-domain (cyan) is ideally positioned to capture signal sequences (orange) as they emerge from the exit tunnel of the ribosome (gray). Structure from PDB: 3JAJ. (C) Co-translational interactions of heteromeric complexes would result in co-localization of their respective mRNAs when two (or more) nascent proteins interact. Once the interaction is established, the polysomal nature of translation re-enforces mRNA co-localization, which would aid further interactions between the nascent chains to mediate assembly.

The best understood example of NC-directed localization of its encoding mRNA is co-translational protein targeting to the ER^109^ (Figure 3A). Around 30% of eukaryotic proteins (roughly 7,000 in humans) are targeted to the ER, nearly all of which arrive there co-translationally as NCs.^110^ This means that the mRNAs coding for those proteins become localized to the surface of the ER. The encoded protein is translocated across or inserted into the ER membrane by machinery that associates with the ribosome. Hence, targeted mRNAs remain at the ER surface, with new ribosomes sustaining this localization before earlier ribosomes finish translation. The molecular details of ER protein targeting are reviewed extensively elsewhere^109^; here, we summarize only those core principles of this classic pathway that likely apply to other examples of NC-directed mRNA localization.

The common feature shared by all ER-targeted NCs is a hydrophobic signal containing 7 to 9 predominantly hydrophobic and helix-compatible residues. Such signals are recognized co-translationally at the ribosome by the SRP, a ribonucleoprotein comprising six proteins assembled on an RNA scaffold.^7^ The M domain of the SRP54 subunit recognizes the signal, whereas another domain of SRP54 binds the ribosome at a site near the mouth of the exit tunnel (Figure 3B). In eukaryotes, promiscuous binding of SRP to the ribosome is antagonized by NAC, a far more abundant factor whose ribosome-binding site overlaps with that for SRP.^111^^,^^112^^,^^113^ Once engaged, SRP targets the ribosome-NC-mRNA complex to a receptor at the ER membrane, where the ribosome is transferred to the Sec61 translocation channel and SRP is recycled back to the cytosol. Thus, specificity for otherwise degenerate hydrophobic signals is achieved in part by a combination of coincidence detection (of both the hydrophobic signal and the ribosome) and antagonism by NAC, whereas high specificity for the destination is imparted by a specific receptor.

The importance of each key element of the ER targeting pathway for accurate protein and mRNA localization has been illustrated from depletion and mutagenesis studies. For example, the hydrophobic signal is necessary and sufficient for localization. In yeast, acute depletion of SRP results in rapid mislocalization of nearly all secretory and membrane protein mRNAs to the cytosol.^114^ By contrast, depletion of NAC in *Caenorhabditis elegans* results in promiscuous SRP-mediated targeting of cytosolic and mitochondrial proteins to the ER.^112^ Thus, NC-directed mRNA localization relies on an NC-encoded signal, a recognition factor, its receptor, and in this particular system, an antagonist to enhance specificity.

These principles can potentially be used for mRNA targeting to other intracellular membranes, although such targeting remains a matter of debate. For example, co-translational targeting of mitochondrial proteins is proposed to be widespread *in vivo*,^106^^,^^115^^,^^116^^,^^117^^,^^118^^,^^119^ despite *in vitro* studies showing that import can occur post-translationally for almost all substrates examined so far.^120^ This controversy is perpetuated by the absence of well-established mitochondrial targeting factors or ribosome docking sites analogous to SRP and Sec61, respectively. Although the yeast-specific alternative β-NAC subunit Btt1 might recognize NCs displaying mitochondrial targeting signals^121^ and engage the mitochondrial protein Om14 as a receptor,^122^^,^^123^ their general roles in targeting are still uncertain.

Nonetheless, it is noteworthy that signals for mitochondrial and chloroplast proteins are often located near the N terminus. This means that they are exposed co-translationally while the remainder of the protein is translated. If they engage organelle translocation machinery prior to termination, the mRNA will become localized. Notably, mRNAs coding for relatively long mitochondrial proteins seem to be preferentially localized and translated on the mitochondrial surface.^116^ Such localization could be sustained and self-reinforcing because new NCs produced from a localized mRNA could more easily engage membrane receptors prior to termination. The generality of this type of mechanism was highlighted by the recent finding that nuclear localization signals of nuclear-localized proteins can be recognized co-translationally, resulting in mRNA delivery to the nuclear pore.^124^^,^^125^ Local translation of these mRNAs adjacent to the nuclear pore may improve the efficiency of nuclear import for the encoded proteins.

## mRNA localization to sub-cytosolic regions

Beyond the surface of membrane-bound organelles, numerous mRNAs are localized to other parts of the cytosol by various mechanisms and for diverse physiologic purposes.^126^ A subset of these mRNAs are thought to be localized co-translationally and seem to rely on the NC.^124^^,^^127^^,^^128^^,^^129^^,^^130^ The transport of mRNA can use multiple mechanisms, depending on the specific example, including use of the actin or microtubule cytoskeleton. Notwithstanding this diversity, the principles are likely to be analogous to those used by the SRP localization system: a recognition element in the NC would be engaged by a specific factor that initiates co-translational localization.

To date, the centrosome and nuclear pore complex are the best examples of NC-directed mRNA localization. Localized translation of subunits comprising these complexes may help avoid inappropriate interactions in the cytosol or facilitate efficient incorporation into pre-existing complexes or scaffolds. The mRNAs for some components of the nuclear pore complex are localized via co-translational NC recognition by nuclear transport factors.^124^ In these examples, the nuclear localization sequence is near the N terminus, allowing sufficient time for localization as a ribosome-NC-mRNA complex. Failure of co-translational localization results in aggregation of unassembled subunits, highlighting the benefit of producing subunits near their site of assembly. Similarly, mRNAs for some centrosome components, such as pericentrin (PCNT) or ASPM, are also co-translationally transported to the centrosome via microtubules at defined stages of the cell cycle.^128^^,^^131^ The mechanism of NC recognition or coupling to the microtubule transport machinery is not understood.

It is noteworthy that the centrosome and nuclear pore complex are both extremely large multi-protein assemblies that are physically constrained within the cell. The assembly of such structures might be particularly challenging, or the consequences of their mis-assembly especially detrimental, to have driven the evolution of mRNA localization mechanisms. Other large complexes might, similarly, utilize NC-directed mRNA localization, an idea worth exploring in future studies. The related notion of co-localizing the mRNAs encoding different subunits of a complex has, indeed, emerged as a mechanism of facilitating assembly, as discussed next.

## Co-localization of mRNAs during complex assembly

The nuclear pore example illustrates how synthesizing different subunits of a protein complex near each other may improve their assembly by minimizing off-pathway interactions. For this to occur, the mRNAs encoding the subunits need to be co-localized. As already described, this can be achieved if the mRNAs or their encoded proteins all share some feature that allows their localization to a particular destination, such as the nuclear pore complex or centrosome. A qualitatively different mechanism involves mRNA co-localization mediated by direct co-translational interactions between their encoded proteins.

The feasibility of co-translational complex formation was shown 60 years ago, when it was observed that a homo-tetramer of prokaryotic β-galactosidase could acquire enzymatic activity while still associated with polysomes.^132^^,^^133^ Similarly, a nascent immunoglobulin heavy-chain molecule was found to co-translationally engage its light-chain partner in eukaryotic cells.^18^^,^^134^ Since these early experiments, it has emerged that co-translational complex assembly is probably widespread.^135^^,^^136^^,^^137^^,^^138^^,^^139^ For example, immunoprecipitation of one subunit of a protein complex often co-precipitates the mRNA of an interacting partner.^124^^,^^140^^,^^141^ This suggests that a nascent subunit can engage its partner during synthesis, a conclusion supported by selective ribosome-profiling experiments in both prokaryotes and eukaryotes.^19^^,^^142^^,^^143^

Such co-translational interactions can occur in one of two ways.^139^ The first way, termed co-post assembly, involves an interaction between the NC for one subunit with another subunit that has already completed synthesis. The second way, termed co-co assembly, involves one NC interacting with the NC of another subunit.^20^ It is easy to appreciate how the second mode of subunit assembly necessarily results in the two encoding mRNAs being co-localized in the cell (Figure 3C). Nevertheless, the first mode can potentially also result in mRNA co-localization under some circumstances. For example, if the “post” half of the interaction is a protein whose mRNA is targeted to a specific part of the cell (e.g., on the ER surface in the case of a membrane protein), co-post assembly will result in the two mRNAs being co-localized. Translation pause sites at strategic locations might provide more time for key co-translational interactions to occur, thereby facilitating assembly.^144^

Once such a co-localization event has occurred, perhaps initially due to stochastic interactions relying on diffusion, it can be maintained by the polysome mode of translation. By the time one ribosome terminates translation to release the NC, other ribosomes would have already engaged in similar co-translational interactions to keep the mRNAs tethered (Figure 3C). In some cases, multiple interacting proteins can be nucleated co-translationally as seems to occur for the yeast SET1C/COMPASS histone methyltransferase complex,^145^ the yeast INO80 chromatin remodeling complex,^140^ subunits of the mammalian transcription factor II D (TFIID),^141^ and the yeast and mammalian nuclear pore complex.^124^^,^^146^^,^^147^ In each of these cases, the NC seems to play a key role in localization of its encoding mRNA.

## Co-translational degradation of tubulin mRNAs

As described above, an NC can trigger degradation of its own mRNA if it causes an irredeemable stall during translation. All of the recognition factors and effectors in stall-triggered degradation are general: the ribosome which detects the stalling element (typically poly-lysine encoded by poly(A)), the sensors of ribosome collisions, and the nucleases that eliminate the mRNA. Hence, the system is general and can act on a wide range of substrates as appropriate for a quality control mechanism. By contrast, a qualitatively different mechanism of NC-triggered mRNA degradation is used for substrate-specific control of mRNA abundance. This mechanism has recently emerged by investigating the basis for a homeostatic feedback pathway termed “tubulin autoregulation” described over 40 years ago.^148^^,^^149^ The pathway is a founding paradigm for selective mRNA decay initiated by its encoded NC, with other examples remaining to be discovered.

**Figure 4:**
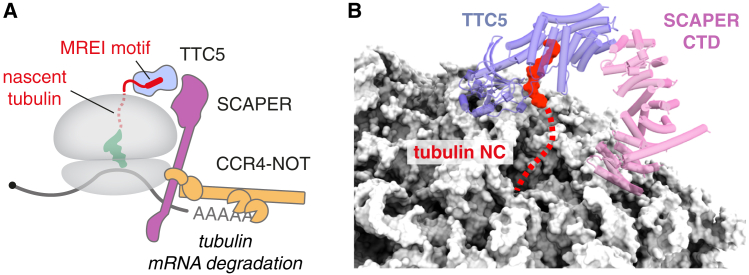
Regulation of mRNA degradation by the nascent chain (A) In the tubulin autoregulation pathway, selective mRNA degradation is achieved by co-translational recognition of an N-terminal sequence motif common to tubulins (MREI for β tubulin). TTC5 is a specific recognition factor that binds both the sequence motif and the ribosome surface. TTC5 recruits the adaptor protein SCAPER, which, in turn, engages the CCR4-NOT deadenylase complex to initiate tubulin mRNA decay. (B) TTC5 binds to the ribosome near the exit tunnel to capture the tubulin nascent chain (NC, red). The adaptor SCAPER is recruited via interactions with both TTC5 and the ribosome (PDB: 8BPO). Only the C-terminal domain (CTD) of SCAPER is shown in this structure; the region that recruits the CCR4-NOT complex is part of an extended helix that was not visualized.

Tubulin autoregulation is a eukaryotic pathway for degrading the mRNAs for both α and β tubulin when cells contain excess unpolymerized αβ-tubulin heterodimers, the building blocks of microtubules (Figure 4). The pathway has long been speculated to be physiologically important because even modest deviations in free tubulin concentration impact microtubule dynamics, which is crucial for cell shape, movement, division, and intracellular transport. Early studies in mammalian cells established that degradation occurs co-translationally and depends critically on the N-terminal tetrapeptide shared among all 18 tubulin isoforms (MREC or MREI for α and β tubulins, respectively).^149^

Recently, tetratricopeptide repeat protein 5 (TTC5) was identified as a ribosome-binding factor that selectively engages the N termini of nascent tubulins.^108^ A structure of TTC5 bound to a tubulin NC revealed that TTC5 binds near the exit tunnel, from where it recruits the adaptor protein SCAPER that also contacts the ribosome^150^ (Figure 4B). Ribosome-bound SCAPER in turn recruits the CCR4-NOT deadenylation complex, which shortens the poly(A) tail of tubulin mRNA to initiate its decay^150^ (Figure 4A). TTC5, SCAPER, and the CCR4-NOT complex are each strictly required for tubulin autoregulation, the absence of which leads to elevated tubulin levels and aberrant chromosome segregation during mitosis.^108^^,^^150^ Human disease mutations of TTC5 and SCAPER lead to defects in neurodevelopment, retinitis pigmentosa, and ciliopathy-related syndromes,^151^^,^^152^ underscoring the physiologic roles for tubulin autoregulation.

This pathway is tightly regulated by poorly understood mechanisms to prevent promiscuous tubulin mRNA degradation. It seems that TTC5 is normally sequestered from engaging the ribosome by some unknown factor unless free tubulin levels rise.^108^ In addition, SCAPER might be sequestered on microtubules and could be regulated throughout the cell cycle.^153^^,^^154^ Finally, ribosome collisions on tubulin mRNAs might modestly impact tubulin mRNA levels via the collision-specific factors 4EHP, GIGYF1, and GIGYF2.^83^ Whether or how these additional factors are regulated by free tubulin levels to modulate autoregulation remains to be investigated. Notwithstanding these unknowns, tubulin autoregulation is the first pathway for which the factors that link direct and specific NC recognition with selective degradation of its encoding mRNA have been established and validated. This system provides a paradigm for investigating whether or how other highly expressed proteins, such as ribosomal proteins or heat-shock proteins,^155^^,^^156^ are similarly regulated.

## Co-translational degradation of orphan-encoding mRNAs

Tubulin autoregulation highlights the existence of co-translational pathways for mRNA abundance control in addition to the well-known co-translational pathways of mRNA quality control. A number of findings suggest that cells contain other co-translational mechanisms to detect and degrade superfluous, but otherwise normal, mRNAs. One example involves the co-translational assembly of mammalian TAF8 with TAF10 to form a subcomplex of TFIID. TAF10 normally interacts with nascent TAF8. In the absence of TAF10, both unassembled TAF8 and its encoding mRNA are degraded by unknown mechanisms.^141^ A similar phenomenon of mRNA destabilization has been reported for the subunits of a voltage-gated potassium channel. In this example, the mRNA for human hERG1a is degraded when its partner hERG1b is eliminated, and vice versa.^157^

In both of these cases, the absence of one subunit of a protein complex destabilizes the mRNA of its interaction partner. The TAF8-TAF10 interaction and hERG1a-hERG1b interaction are both thought to be initiated co-translationally. This raises the possibility that an NC that fails to make a key co-translational interaction important for its biogenesis triggers degradation of its encoding mRNA. Two other examples support this general idea. First, the mRNA for a signal sequence-containing protein is selectively degraded when the targeting factor SRP was depleted.^158^ Second, the mRNAs for two ribosomal proteins in yeast (Rpl3 and Rpl4) are degraded when their cognate chaperones were absent.^156^ The common feature shared by all of these examples is that the degraded mRNA encodes for a protein that would be orphaned (i.e., unassembled or mislocalized) if it were produced. Although orphans are typically eliminated post-translationally to minimize their detrimental effects,^159^ degradation of their corresponding mRNA may be important in particular cases.

**Figure 5:**
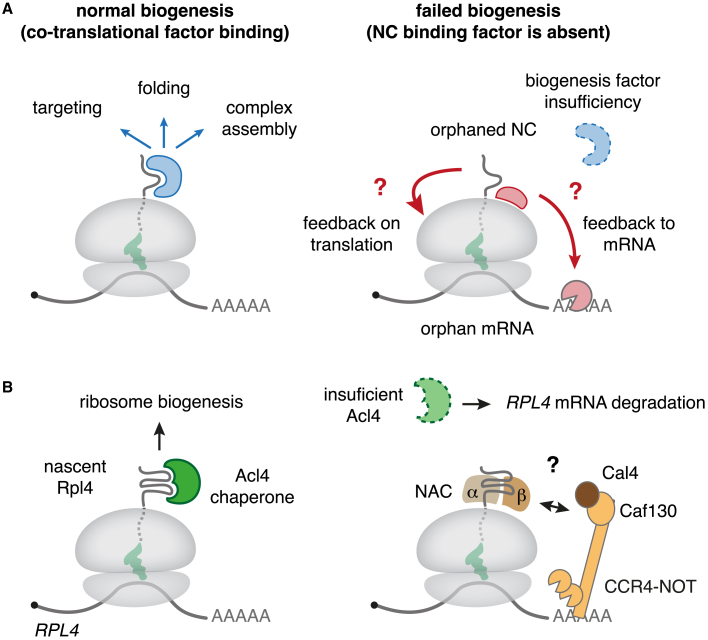
Model for biogenesis-coupled mRNA decay (A) General model for how failures during co-translational biogenesis could lead to mRNA decay. Left: under normal biogenesis conditions, many nascent chains require co-translational interaction partners (blue) for productive targeting, folding, or complex assembly. Right: if a cognate interaction partner is limiting, the nascent chain is orphaned, and the ribosome or NC becomes accessible for binding by other factors (red) that might act to inhibit translation or trigger mRNA decay. (B) Schematic of the *Saccharomyces cerevisiae* autoregulation pathway for the *RPL4* mRNA encoding for Rpl4, a ribosomal protein. Left: normally, the dedicated chaperone Acl4 engages with nascent Rpl4 to chaperone it and enable productive ribosome biogenesis in the nucleus. Right: when Acl4 is limiting, the *RPL4* mRNA is destabilized by a mechanism that requires a general ribosome-binding factor called NC-associated complex (αβ-NAC), the CCR4-NOT complex with its Caf130 subunit, and Cal4. The molecular mechanism by which these factors mediate selective mRNA degradation is not clear but might involve direct CCR4-NOT complex recruitment via NAC.

The observations above suggest an intriguing model in which co-translational degradation is the default pathway for some mRNAs unless this fate is rescued by a biogenesis factor that engages the encoded NC (Figure 5A). Such a mechanism would ensure that only mRNAs coding for biogenesis-competent proteins are stable. Although attractive, support for this idea is modest. The pathway of mRNA decay for *TAF8* or the hERG subunits has not been explored. In the case of SRP-dependent substrates, AGO2 was initially implicated but later called into question.^158^^,^^160^ The best evidence has come from the yeast ribosomal protein system, where a recent study identified both the NC element and a subset of factors that trigger mRNA decay.

Both Rpl3 and Rpl4 co-translationally engage their respective chaperones, in the absence of which these ribosomal protein mRNAs are degraded (Figure 5B).^156^ Degradation was found to depend on NAC and the CCR4-NOT complex with the adaptor protein Caf130. In the case of Rpl4, an additional Caf130-associated protein, Cal4, was also needed. Mutagenesis experiments showed that the peptide motifs on Rpl3 and Rpl4 required for mRNA degradation are adjacent to the chaperone binding sites. These observations were placed into a model where these motifs are recognized by NAC, which recruits the CCR4-NOT complex to initiate mRNA degradation.^156^ This fate can be avoided by co-translational chaperone engagement, which would sterically occlude the NAC-binding motif.

Because NAC and the CCR4-NOT complex are conserved, widely expressed, and abundant, this mechanism could potentially apply to other examples of orphan-encoding mRNAs. The key requirement would be a NAC-binding motif at or near a site that would be occluded if co-translational biogenesis proceeds normally. Indeed, SRP triggers NAC displacement from its binding site on the ribosome,^113^^,^^161^ perhaps explaining why SRP’s absence results in degradation of a signal sequence-encoding mRNA. This speculative model warrants careful scrutiny in future work.

A non-mutually exclusive and equally speculative model is one where translational arrest sequences are very widespread but constantly obscured by the weak pulling force imparted by factor binding to the NC just outside the ribosome exit tunnel. In this model, stalling and ensuing mRNA decay would be the default, being avoided under normal conditions by the constant availability of chaperones and other biogenesis factors. In support of such a model, it was observed that after acute and severe protein misfolding stress when chaperone activity is limiting, ribosomes on a wide range of mRNAs stall early during synthesis at a point when the NC is just emerging from the exit tunnel.^97^^,^^98^ This suggests that chaperones normally aid elongation, perhaps by providing some tension in the NC to avoid a sub-optimal geometry of the peptidyl-tRNA at the PTC.

## Conclusions

The transient proximity of an NC with its encoding mRNA affords numerous opportunities for the NC to regulate the stability, processing, localization, and translation of the associated mRNA. A few of these mechanisms, such as regulation by bacterial arrest peptides and NC-mediated mRNA targeting by SRP, are well established and have generally satisfying molecular explanations. A few others, such as non-stop mRNA decay and tubulin autoregulation, are only now emerging in mechanistic detail. Currently, most other potential examples are poorly understood, being limited to initial descriptions of the phenomenon with few, if any, molecular insights. By highlighting these observations and articulating some potential, albeit speculative, molecular models, we aim to stimulate research in this area. The central role of translation and protein biogenesis in nearly all areas of biology suggests that our understanding of the links between the nascent protein and its mRNA is at an early stage. We therefore anticipate many exciting discoveries in this newly developing area of post-transcriptional mRNA regulation.

With a greater mechanistic understanding of this type of regulation, novel approaches to therapeutic intervention may emerge. Specific NCs or their *trans*-acting factors might be amenable to selective targeting by drugs to control the usage and fate of an mRNA. Translational regulation plays roles in physiologic processes as diverse as memory formation and responses to stress,^162^^,^^163^ whereas translational dysregulation features prominently in many diseases, including cancer.^164^^,^^165^ Furthermore, mRNA vaccines are gaining importance for treatment of infectious diseases, cancer, and other conditions.^166^^,^^167^ A mechanistic understanding of the interplay of any given NC with its encoding transcript may facilitate the optimal design of therapeutic mRNA vaccines.
